# The Influence of HCl Concentration on the Rate of the Hydrolysis–Condensation Reaction of Phenyltrichlorosilane and the Yield of (Tetrahydroxy)(Tetraphenyl)Cyclotetrasiloxanes, Synthesis of All Its Geometrical Isomers and Thermal Self-Condensation of Them under “Pseudo”-Equilibrium Conditions

**DOI:** 10.3390/molecules26144383

**Published:** 2021-07-20

**Authors:** Irina M. Petrova, Yury I. Lyakhovetsky, Nikolai S. Ikonnikov, Nataliya N. Makarova

**Affiliations:** A.N. Nesmeyanov Institute of Organoelement Compounds of the Russian Academy of Sciences, 28 Vavilov St., 119991 Moscow, Russia; impetr2013@yandex.ru (I.M.P.); yulyakh@ineos.ac.ru (Y.I.L.); ikonns@ineos.ac.ru (N.S.I.)

**Keywords:** hydrolysis-condensation, self-condensation, “pseudo”-equilibrium conditions, phenyltrichlorosilane, isomers of (tetrahydroxy)(tetraphenyl)tetrasiloxane, cage-like compounds, NMR, APCI-MS

## Abstract

The rate of hydrolysis–condensation reaction of phenyltrichlorosilane in water-acetone solutions and the product yields were shown to significantly depend on the concentration of HCl (C_HCl_) in the solutions. The main product of the reaction was all-*cis-*(tetrahydroxy)(tetraphenyl)cyclotetrasiloxane. This was different from the earlier published results of analogous reactions of *m*-tolylSiCl_3_, *m*-ClPhSiCl_3_, and *α*-naphtylSiCl, in which some products of other types were formed. For example, *trans*-1,1,3,3-tetrahydroxy-1,3-di-*α*-naphtyldisiloxane was obtained in the case of *α*-naphtylSiCl_3_. All-*cis-*(tetrahydroxy)(tetraphenyl)cyclotetrasiloxane was treated in acetone with HCl to give the other three geometric isomers (*cis-cis-trans*-, *cis-trans-*, and all-*trans-*). The thermal self-condensation of these four isomers under “pseudo”-equilibrium conditions (under atmospheric pressure) was investigated in different solvents, in quartz or molybdenum glass flasks. The compositions of the products were monitored by APCI-MS and ^29^Si NMR spectroscopy. It was shown that all-*cis*- and *cis-cis-trans*-isomers in toluene or anisole mostly gave the cage-like Ph-T_8,10,12,14_ and uncompleted cage-like Ph-T_10,12_OSi(HO)Ph compounds. In contrast to these two isomers, the *cis-trans*–isomer in toluene mainly formed dimers with the loss of one or two molecules of water. However, in acetonitrile, significant amounts of Ph-T_10,12_ and Ph-T_10,12_OSi(HO)Ph species were formed along with the dimers. All-*trans*-isomer did not enter into the reaction at all.

## 1. Introduction

Polyorganosilsesquioxanes (POSSO), compounds with the formula (RSiO_1.5_)_n_, are the object of comprehensive studies since they have unique thermal stability, low dielectric constants and hygroscopicity, can serve as binders for ceramics, and form thin films and microporous solid materials. POSSO formed as cyclolinear, cage-like species with different degree of completion, and as branched polymers.

Data on hydrolysis-condensation and -polymerization of organotrichloro- and organotrialkoxysilanes at different concentrations in solvents, different temperatures, and in the presence of potassium hydroxide were summarized in a review [1]. They included the change in properties of POSSO in dependence on the molecular weights, *α* values in the Mark-Houwink equation, solubility, and weight losses at high temperatures. In another review, information was given on the synthesis, structure, and physical and chemical properties of polyhedral organosilsesquioxanes [2]. Also, therein, the effects of initial functional monomer concentrations in the solutions, the nature of substituents and functional groups, and the type of catalyst upon the reaction rates, the degree of oligomerization, and the yields of cage-like compounds were reviewed. Cage-like species with carbofunctional groups were synthesized by the hydrosilylation of the corresponding carbofunctional olefins with octahydridsilsesquioxane as well as with sila-functional cubes [3]. Significant progress was made in the synthesis of molecularly ordered nanoporous materials by the controlled binding of cage siloxanes based on their self-assembly due to H-bondings and their regioselective modification with silanol or alkoxy groups [4].

Completed and incomplete cage-like compounds were used as mono- and multifunctional precursors for the synthesis of comb-like and branched copolymers [5]. Polyhedral compounds appear to be among the most interesting precursors for use as building blocks for constructing high-performance organic optoelectronic materials: electroluminescent and electrochromic devices, and liquid crystals [6]. POSSO with organic groups such as alkyl, aryl, alkoxy or hydride, those obtained via the hydrolysis and polycondensation of trialkoxy- or trichlorosilane monomers, contain reactive or non-reactive organic groups. Various organosilsesquioxane (OSSO)-based hybrid nanomaterials were examined with regard to their application as polymer nanocomposites, catalysts, sensors, and for medical purposes [7].

In recent years, new approaches to the synthesis of both POSSO and PPSSO (polyphenylsilsesquioxanes) have been tested, namely, the use of different building blocks such as (tetrahydroxy)(diphenyl)disiloxane [8], tetrametoxydisiloxane [9], and isomers of (tetrahydroxy)(tetraphenyl)cyclotetrasiloxane in the hydrolysis-polycondensation reactions in order to obtain stereoregular cyclolinear POSSO or PPSSO (See also [10,11,12,13]). The hydrolysis–condensation reaction of phenyltrimetoxysilane in dilute solutions of various solvents resulted in dodecaphenylsilsesquioxane (Ph-T_12_). An increase in concentration of the former led to cyclolinear PPSSO [14,15]. Octaphenylsilsesquioxane (Ph-T_8_) was obtained when the condensation of all-*cis*-(tetrahydroxy)(tetraphanyl)cyclotetrasiloxane was implemented at r.t. in the absence of a catalyst [16]. Octaarylsilsesquioxanes with reactive or non-reactive organic groups were synthesized from cyclotetrasiloxanetetraols and their sodium salts in the presence of ammonium catalysts [17]. Unsymmetrical silsesquioxane double-deckers of new types [17,18,19,20,21,22], the basket [23] and butterfly types [24], or siloxane cages were reported to be obtained by simple hydrolytic condensations of two cyclosiloxanes with an organomonosilane. They can be building blocks for diverse applications since materials based on them should have low dielectric constants and excellent thermal stability.

Hybrid cage-type organosiloxane oligomers can be formed by hydrolysis and polycondensation of bis(trialkoxy)silane [(EtO)_3_Si-CH_2_-Si(OEt)_3_] in the presence of a tetramethylammonium salt [25].

Recently, we studied how the concentration of HCl in solutions, and characteristics of substituents, affected the results of the hydrolysis–condensation reaction of three aryltrichlorosilanes, *m*-tolylSiCl_3_, *m*-ClPhSiCl_3_, and *α*-naphtylSiCl_3_. The alterations in composition and structure of intermediates and final products occurred due to variation of these factors were followed by atmospheric pressure chemical ionization mass spectrometry (APCI-MS) and^29^Si NMR [26]. One of the purposes of the present study was to compare the results of the above study with those of the analogous reaction of PhSiCl_3_.

## 2. Results and Discussion

**Figure 1:**
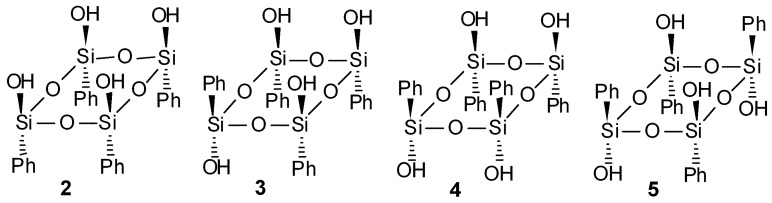
The geometric isomers of (tetrahydroxy)(tetraphenyl)cyclotetrasiloxane.

We have now studied the hydrolysis–condensation reaction of phenyltrichlorosilane (**1**) at different concentrations of HCl (C_HCl_) in water-acetone solutions. Then, the thermal self-condensations in “pseudo”-equilibrium conditions (under atmospheric pressure) of four isomeric tetrahydroxy-(tetraphenyl)cyclotetrasiloxanes: all-*cis*- (**2**), *cis-cis-trans*- (**3**), *cis-trans*-(**4**), and all-*trans*-(**5**) (Figure 1) have been performed.

**Figure 2:**
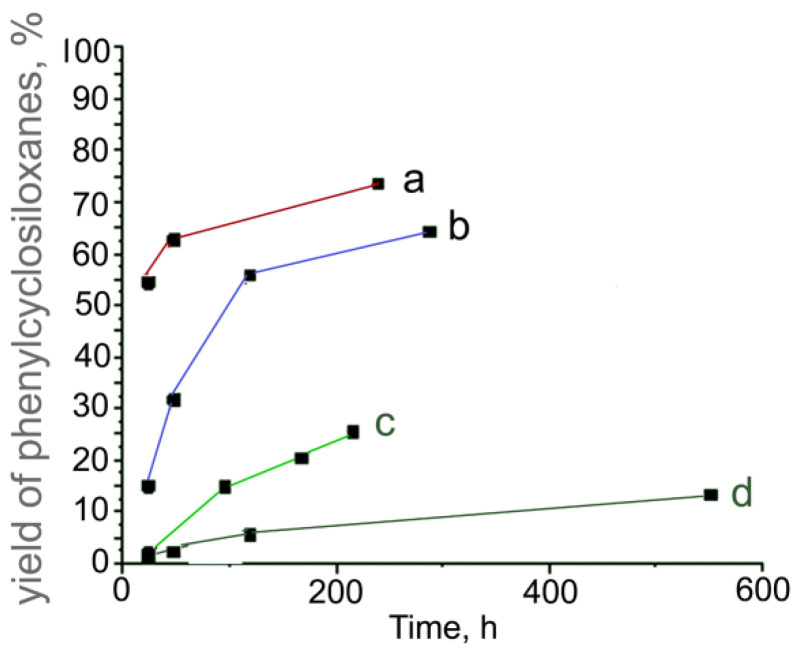
Dependence of the yields of phenylcyclosiloxanes from the hydrolysis–condensation reaction of **1** on time at different concentrations of C_HCl_ in water-acetone solutions: C_HCl_ = 0.26 (**a**); 0.15 (**b**); 0.055 (**c**), and 0.032 mol L^−1^ (**d**).

All reactions were monitored with APCI-MS and ^29^Si NMR spectroscopy. The reactions of **1** were conducted at 4 °C with the concentration of HCl between 0.032 and 0.26 mol L^−1^. The yields of the products were determined in the interval of 24–570 h. The reaction conditions were selected to be similar to those previously published for three aryltrichlorosilanes [26], to compare the composition and structure of intermediates and final products. Figure 2 shows these yields of the products that precipitated from the solution depending on the reaction time. As can be seen from Figure 2, the rate of the reaction and final yields of the products decreased with the decrease in acidity (See Note 1 in the Supplementary Materials (SM)).

**Figure 3:**
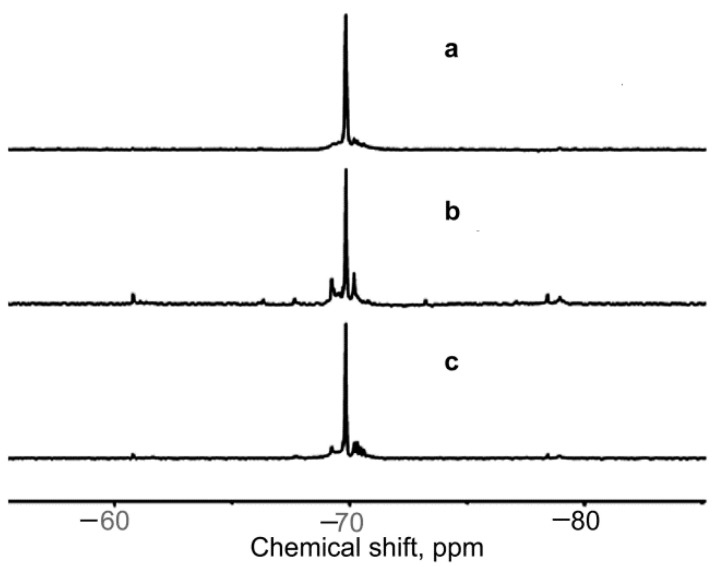
^29^Si NMR spectra of phenylcyclosiloxane compounds from the reaction of **1** carried out for 24 h at C_HCl_ = 0.15 mol L^−1^ (**a**); 120 h at C_HCl_ = 0.15 mol L^−1^ (**b**), and 24 h at C_HCl_ = 0.26 mol L^−1^ (**c**).

Figure 3 presents the ^29^Si NMR spectra of phenylcyclosiloxane compounds obtained at C_HCl_ = 0.15 and 0.26 mol L^−1^, separated from the reaction mixture after the 24 h reaction. Samples of them were dissolved in acetone-*d*_6_ and subjected to ^29^Si NMR.

A number of signals at −69.0~−71.0 ppm, with one at −69.79 ppm being greatly predominant, were observed for the products obtained at C_HCl_ = 0.15 and 0.26 mol L^−1^, while any signals in the regions of −50.0 and −80.0 ppm were absent. This evidenced that the products had only one OH group at each silicon atom. All of this was valid for compounds from the reaction conducted at C_HCl_ = 0.055 mol L^−1^. The TLC tests of all the products with the toluene:diethyl ether = 4:1 eluent gave a value of R_f_ = 0.05 that was consistent with previously published data for all-*cis*-isomer of (tetrahydroxy)(tetraphenyl)cyclotetrasiloxane (**2**) [27,28]. The small signals around the intensive one most likely belonged to other isomers of (tetrahydroxy)(tetraphenyl)cyclotetrasiloxane. The calculation of the ratio of the integral intensity of the main signal to the sum of it and the integral intensities of the small signals around it made for spectrum “c” in Figure 3 showed that the content of isomer **2** in the mixture of isomers ought to be higher than 95%.

It was of interest to compare the results of the hydrolysis-condensation reaction of PhSiCl_3_ with those of hydrolysis-condensations of *m*-tolylSiCl_3_, *m*-ClPhSiCL_3_, and *α*-naphtylSiCl_3_ reported in [26].

For all these compounds, the rates of the reactions and the yields of products increased with the increase in the acidity of the reaction mixtures.

However, there were differences in the types of the products.

Thus, the ^29^Si NMR spectra of the products (Figure 3) showed that, in the hydrolysis–condensation reaction of **1**, at different C_HCl_ in the solutions, isomer **2** formed mostly, and other isomers formed in negligible amounts or isomerization of **2** to them occurred to a very small extent, even if the reaction was conducted for 120 h. Compound **2** was obtained in the 73.6% yield (See Section 3.3.1).

The ^29^Si NMR spectra in Figure 3 (the spectra of the precipitates when the reaction was performed at the C_HCl_ = 0.055 mol L^−1^ were similar) were compared with those for the hydrolysis–condensation of *m*-tolylSiCl_3_ implemented at the C_HCl_ = 0.054 mol L^−1^ and reported in [26]. In the former case, as was told above, the intensive singlet of **2** at −69.79 ppm was present in the spectra. At the same time, in the latter case, two singlets at −69.98 and −70.49 ppm (these values were more accurately measured than those given in [26]) were first present in the ^29^Si NMR spectrum, the upfield singlet being of an even greater intensity than the downfield one. Then the intensity of the upfield signal decreased in time until it completely disappeared, and only the downfield singlet remained in the spectrum. The upfield signal was ascribed to the *cis*-*trans*-isomer of (tetrahydroxy)(tetra-*m*-tolyl)cyclotetrasiloxane (In [26], the *cis*-*trans*-isomer was mistakenly named as the *cis*-*trans*-*cis*-isomer) which graded into all *cis*-isomer, that gave the downfield signal at −69.98 ppm.

Two singlets in the region of −71.3 to −72.3 ppm (the more accurate measurements gave values −71.55 and −71.78 ppm) were found in the ^29^Si NMR spectrum of the precipitate from the reaction mixture of the hydrolysis–condensation of *m*-ClPhSiCl_3_ carried out at C_HCl_ = 0.032 mol L^−1^ for 48 h. They were ascribed to the all *cis*- and *cis*-*trans*-isomers of (tetrahydroxy)(tetra-*m*-chlorophenyl)cyclotetrasiloxanes, respectively [26].

Two singlets at −60.68 and −61.64 ppm were present in the ^29^Si NMR spectrum of the precipitate from the reaction of the hydrolysis–condensation of *α*-naphtylSiCl_3_ conducted at C_HCl_ = 0.15 mol L^−1^. Most likely, they belonged to isomers of 1,1,3,3-tetrahydroxy(1,3-di-*α*-naphtyl)disiloxane.

**Scheme 1:**
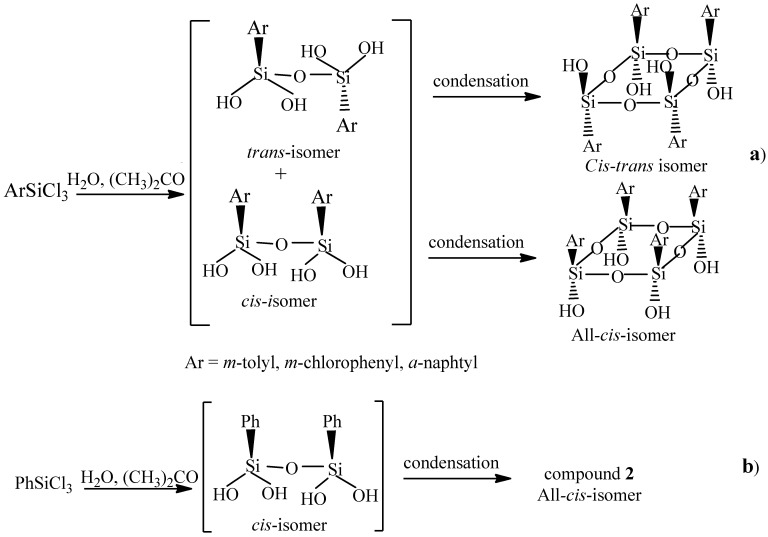
The ways of formation of stereoisomers of (tetrahydroxy)(tetraaryl)cyclotetrasiloxanes in the hydrolysis–condensation of *m*-tolyl-, *m*-chlorophenyl-, or *α*-naphtyltrichlorosilane (**a**) and phenyltrichlosilane (**b**).

What is the reason for such different steric results for the reactions of **1** and its aryl analogs? Probably, in the last three cases, *trans-*isomers of 1,1,3,3-tetrahydroxy(1,3-diaryl)disiloxanes formed as intermediates along with the *cis*-isomers. They seem to be more stable than the *cis*-ones since the *m*-tolyl, *m*-ClPh, and *α*-naphtyl substituents are more bulky than the Ph one, and the reaction partly occurred through them. The condensation in the case of **1** occurred completely via the formation of the *cis*-isomer as an intermediate (Scheme 1).

As an indirect evidence in support of Scheme 1, it is necessary to mention that the *trans*-isomer of 1,1,3,3-tetrahydroxy-1,3-di-*α*-naphthyldisiloxane was obtained in 48% yield by the hydrolysis–condensation reaction of *α*-naphtylSiCl_3_ carried out at C_HCl_ = 0.15 mol L^−1^ for 190 h. It was characterized by the XRPD method [26].

Thus, in the case of *m*-tolylSiCl_3_, though the analog of 2, all *cis*-(tetrahydroxy)(tetra-*m*-tolyl)cyclotetrasiloxane was obtained in the 30.8% yield, the *cis*-*trans*-isomer formed in significant amounts in the course of the reaction. It was then graded into all *cis*-isomer. This was not characteristic of the reactions of **1**.

Besides, at a greater acidity, a mixture containing many *m*-tolylcyclosiloxanes was obtained in the course of the 24 h reaction of *m*-tolylSiCl_3_, as was shown by PI APCI-MS. 

In the case of *m*-ClPhSiCL_3_, two isomers of (tetrahydroxy)(tetra-*m*-chlorophenyl)cyclotetrasiloxane were obtained by the hydrolysis-codensation reaction of it: the all-*cis* one in the yield of 19% and the *cis*-*trans*-isomer in the yield of 24.9%. Apart from this, the formation of poly-*m*-chlorophenylsilsesquioxanes in the course of the reaction was displayed by ^29^Si NMR.

The *trans*-isomer of 1,1,3,3-tetrahydroxy-1,3-di-*α*-naphtyldisiloxane was obtained in the 48% yield by the hydrolysis–condensation reaction of *α*-naphtylSiCl_3_. Besides, PI APCI-MS showed some other condensation products (e.g., *α*-naphtyl-T_8_ and *α*-naphtyl-T_10_) to be present in the reaction mixtures.

As it was told above, other isomers apart from **2** were formed in negligible amounts in the course of the hydrolysis-condensation reaction of **1.** However, it was earlier reported that **2** underwent isomerization to the other isomers in a dilute acetone solution if hydrochloric acid or methylchlorosilanes were added to the solution [27,28,29].

Recently, the stereoisomerization of all *cis*-[*iso*-C_4_H_9_Si(OH)O]_4_ in an acetone solution under the action of HCl taken in concentrations from 0.01 M to 5 M was studied. It was found that after 10 min, regardless of the C_HCl_, three isomers were formed and the ratio between the four isomers changed insignificantly with the change in C_HCl_ [30].

We prepared these other isomers of **2** with the protocol reported in [28].

The isomers were separated by fractional crystallization and their identification was first made by the R_f_ values and IR spectra [27]. According to the ^29^Si NMR spectral data, the presence of 5% of any isomer **2**–**5** in solution could easily be determined [28]. Thus, we used ^29^Si NMR for the additional identification of the isomers. With that, it was taken into account that the chemical shifts (δ) for stereoisomers **2**–**5** changed depending on the concentration and composition of the mixture, however the differences (Δδ) between three singlets of isomer **2**, **4**, and **5** were constant, whereas isomer **3** gave three singlets with the ratio of 1:2:1. APCI-MS was also employed for this purpose in the cases of **2** and **3** (see below). Previously, structures of **2** and **5** were confirmed by X-ray powder diffraction and single crystal analysis [31] and [10], respectively

Stereoisomers **2**–**5** were used as the starting compounds for the condensations carried out under “pseudo”-equilibrium conditions (under atmospheric pressure). As it was told above, APCI-MS and ^29^Si NMR were employed for monitoring the reactions.

**Figure 4:**
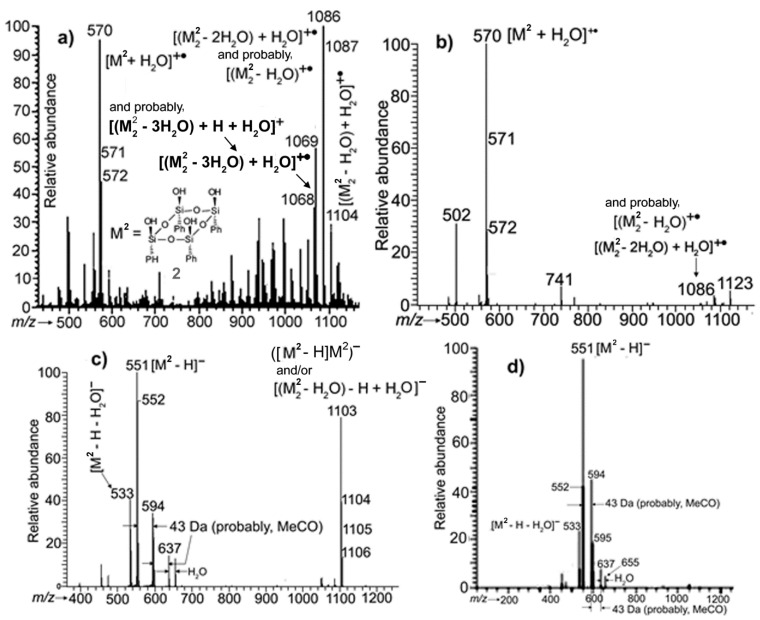
APCI mass spectra of isomer **2**: (**a**,**b**)—recorded in the PI mode when the concentration of **2** in analyte was greater and less, respectively; (**c**,**d**)—in the NI mode, analogously.

As mentioned above, the APCI-MS was also used in the identification of isomers **2** and **3**. However, these compounds, as well as **4** and **5**, could enter into the self-condensation reaction under conditions of APCI–MS. Because of this, the PI (positive ion mode) and NI (negative ion mode) APCI mass spectra of **2** and **3** were thoroughly analyzed and additional spectra were recorded. It was believed that this would allow us to understand what results corresponded to the reactions in flasks rather than in the mass spectrometer. Samples of **2** and **3** were dissolved in acetonitrile before introduction in the instrument. *A priori*, the results could depend on the concentration of **2** or **3** in this solvent. That is why the spectra of **2** were recorded at different dilutions. Figure 4 shows these mass spectra.

When the concentration of **2** was relatively high, the PI APCI mass spectrum displayed a peak at *m/z* 570 due to ion [M^2^ + H_2_O]^+•^ (See Note 2 in the SM), whereas peaks of [M^2^]^+•^ and [M^2^ + H]^+^ were virtually absent (Figure 4a). In the spectrum, the maximal ion peak proved to be that of a compound formed owing to the uncompleted condensation of two molecules of **2** with a water molecule added in the mass spectrometer ([(M^2^)_2_ − 2H_2_O) + H_2_O]^+•^ at *m/z* 1086). Ion [(M^2^)_2_ − H_2_O) + H_2_O]^+•^ at *m/z* 1104 and ions from a dimer formed due to the loss of three water molecules in the course of the condensation (also with the addition of H_2_O during the analysis) were also present (See Note 3 in the SM). 

However, when the analyte was significantly diluted with acetonitrile, the self-condensation of **2** was almost suppressed, and the mass spectrum displayed the [M^2^ + H_2_O]^+•^ ion peak being predominant (Figure 4b).

In the NI APCI mass spectrum of the former variant of analyte, ion peaks of anions [M^2^ − H]^−^ at *m/z* 551 and [(M^2^ − H)M^2^]^−^ or [((M^2^)_2_ − H_2_O) − H + H_2_O]^−^ at *m/z* 1103 were present. For the latter (diluted) analyte, the peak of the dimer was absent (Figure 4c,d, respectively; also see Note 4 in the SM).

For compound **3**, the most abundant ion peak in PI APCI mass spectra turned out to be one of radical cation [M^3^ + H_2_O]^+•^, and ion peaks due to higher molecular mass compounds were more abundant than in the case of **2** (See Figure S1 in the SM where ions recorded by APCI-MS are specified).

The results obtained showed that, when analyzing the reaction mixtures of the self-condensation of species **2**–**5** by APCI-MS, if the starting isomer still remained in the mixture, the samples for analysis should sufficiently be diluted to avoid additional reactions occurring immediately inside the mass spectrometer.

**Figure 5:**
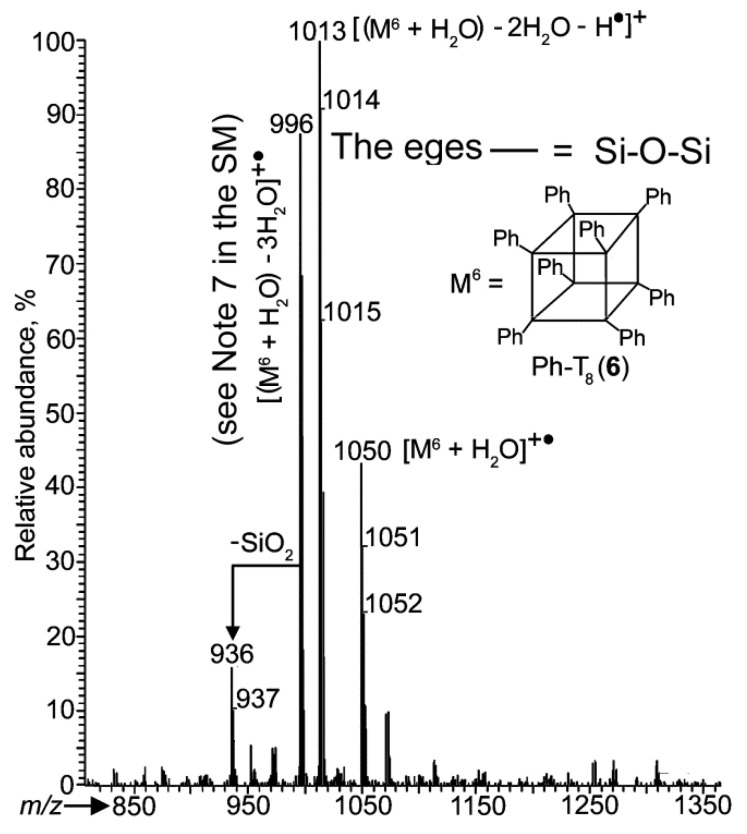
PI APCI mass spectrum of compound **6**.

**Figure 6:**
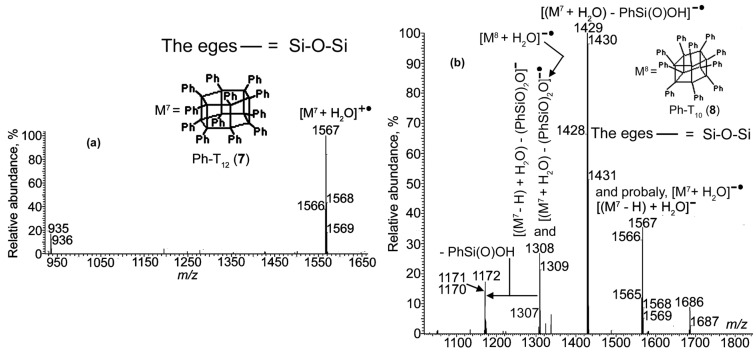
APCI mass spectra of compound **7** recorded in (**a**) the PI mode and (**b**) in the NI mode.

Moreover, polyhedral compounds: octaphenylsilsesquioxane (Ph-T_8_) and dodecaphenylsilsesquioxane (Ph-T_12_), (**6** and **7**, respectively) were awaited to be formed as products of condensations of species **2**–**5**. Though APCI-MS is considered as a rather mild method of ionization, fragmentations of ions could occur, especially as “in source collision induced dissociations”, owing to the construction peculiarity of the instrument [32,33]. Thus, it was also interesting to obtain the APCI mass spectra of **6** and **7** and estimate how the fragmentations of ions of these compounds could hinder understanding what products and intermediates formed in the course of the reactions in flasks. Compounds **6** and **7** were obtained by the corresponding protocols described in [34] and [14], respectively. Their APCI mass spectra are presented in Figure 5 and Figure 6.

Figure 5 and Figure 6 displayed fragment ions that exist in the PI APCI mass spectra for **6** and in the NI mode for compound **7**. As underlined above, this should be taken into account when analyzing the reaction mixtures with these methods.

The spectra also demonstrated both these compounds to be registered in the PI mode (analogously to **2** and **3**) as hydrates of their ions (see the last sentence of Note 2 in the SM) with the molecular ions being absent. Spectrum **6b** showed that all of this was valid for compound **7**, even in the NI mode.

**Figure 7:**
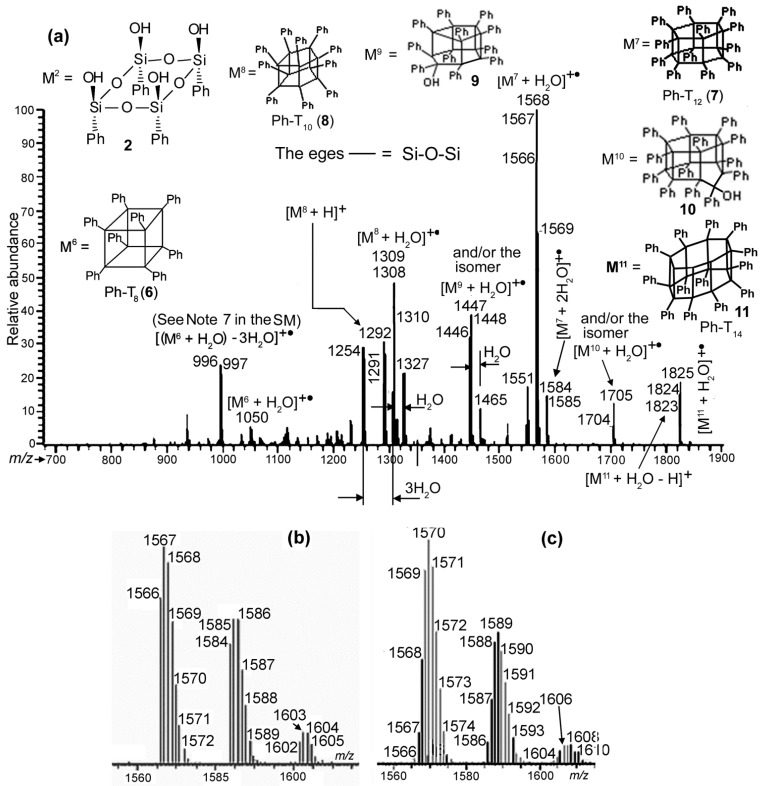
(**a**) PI APCI mass spectrum of the precipitate from the reaction mixture of the self-condensation of compound **2** carried out in a quartz flask in toluene at 110 °C for 120 min, (**b**,**c**)—The *m/z* 1555–1615 segments of the spectra recorded when H_2_O and D_2_O were simultaneously syringed into the mass spectrometer, respectively.

The reaction of condensation of compound **2** was first performed in toluene solution in a quartz flask at 110 °C for 120 min (Note 5 in the SM). The PI APCI mass spectra of the precipitates from the reaction mixture were taken after 30, 45, and 120 min periods. The spectra of the precipitates obtained after the 45 and 120 min periods are given in Figure S2 in the SM and Figure 7a, respectively. They do not contain peaks at *m/z* 570, characteristic of the starting compound. This means that they show products of the solution reaction rather than those of the reaction that occurred in the mass spectrometer.

Of interest are groups of the most intensive peaks at *m/z* 1566, 1446, 1308, 1291, 1254, and 996 (the peaks of the nominal mass ions) in Figure 7a. The 1566 group obviously belongs to the hydrate of compound **7** (Ph-T_12_), and the lower mass ions are not its fragment ions. This comes from the fact that the fragmentations were not observed in the PI APCI mass spectrum of pure **7** (See Figure 6a). Moreover, the ms^2^ spectrum of the 1568 Da ion supported this. Also, the ions of the 1308 ion group are not the fragment ions of the 1446 one, since the ms^2^ spectrum of the 1446 Da ion showed it. Thus, the 1446 and 1308 ion groups correspond to the hydrates of products of the reaction in the flask. The latter is obviously present due to the hydrate of compound **8** (Ph-T_10_, see Figure 7a). The former group can be attributed to the hydrates of species **9**, (Ph-T_10_OSi(OH)Ph, also see Figure 7a) and/or the isomeric one where the OSi(OH)Ph group is connected to the polyhedral cage with breakage of a vertical edge rather than one in a pentagon. The ion with the nominal mass 1291 Da could be the protonated molecule of **8**, and/or a fragment ion of [M^8^ + H_2_O]^+•^ formed via the loss of ^•^OH from it. However, the ms^2^ spectrum of the 1308 Da ion showed the fragmentation producing the 1290 Da ion (the loss of a molecule of water). Thus, the 1291 Da ion appears to be the protonated molecule of the reaction product **8**.

One more product was found, compound **6** (Ph-T_8_) registered as the molecular radical cation of its hydrate, [M^6^ + H_2_O]^+•^. The 996 Da ion appeared to be its fragment ion formed due to the loss of three water molecules from this radical cation. This found support from the finding that this ion was present in the PI APCI mass spectrum of pure **6** (Figure 5). A similar process was observed for the 1308 Da ion to give the 1254 Da ion.

Two other products were detected. Their possible structures are depicted in Figure 7 under numbers **10** (Ph-T_12_OSi(OH)Ph) and **11** (Ph-T_14_)).

Two comparative experiments were made with the precipitate from the 120 min reaction mixture. Samples of it in acetonitrile were introduced in the mass spectrometer, either water or heavy water (D_2_O) being introduced simultaneously. Figure 7b,c give the PI APCI mass spectra in the region of the molecular ions of the compound **7** hydrates for the H_2_O and D_2_O cases, respectively. As expected, in the second spectrum, the peak of the ion with the nominal mass of 1566 Da shifted by two units to that of 1568 Da, indicating the addition of a D_2_O molecule. An interesting fact was also observed: a peak at *m/z* 1584 of the dihydrate was registered at *m/z* 1588. This indicated the addition of two water or heavy water molecules to the molecular ion of **7** ([M^7^]^+•^) in the mass spectrometer (see Note 6 in the SM). A group of peaks beginning with that at *m/z* 1602, belonging to the trihydrate, were found in spectrum 7a. Most likely, the third water molecule added to [M^7^]^+•^ in the mass spectrometer; the abundances of the corresponding peaks in spectrum 7b, however, were too small to make the correct conclusion.

The mass spectra of the solution and the precipitate from the same time reaction mixture were virtually similar. This proved to be so for the reactions of **2**–**4** carried out under any other conditions employed.

As mentioned before, Figure S2 in the SM and Figure 7a present the PI-APCI mass spectra recorded after 45 and 120 min of heating of compound **2** at 110 °C in toluene, respectively. They reflected changes in the reaction mixture in time: a decrease in products of the condensation of two molecules of **2** (peaks of the nominal mass ions at *m/z* 996 and 1050) and an increase in those of three and four molecules (peaks at *m/z* 1254, 1291, 1308, 1446, 1566, 1584, and 1704, 1824, respectively).

It was previously reported that, when implementing the zone melting of a mixture of isomeric cyclosiloxanes in a molybdenum glass ampoule, a polymerization process was observed. The authors believed this to occur owing to the breakage of the cyclosiloxane rings catalyzed by metal ions present in the glass [35]. Bearing this in mind, we carried out the reaction of **2** in a molybdenum glass flask in toluene at 110 °C for 120 min. In the PI-APCI mass spectrum of the precipitate (Figure S3 in the SI), all ion peaks that were found in the corresponding spectrum of the precipitate from the reaction in the quartz flask were present. However, the ion peaks due to product **9** turned out to be significantly greater in abundances. As mentioned above, it probably could be owing to the catalysis by metal ions present in molybdenum glass, however, further thorough experiments are required to prove this.

The ^29^Si NMR spectrum of this precipitate showed two groups of signals, at −68.0~−71.0 and −76.0~−79.5 ppm. The former contained signals of low intensities belonging to the –OSi(OH)Ph groups. The latter consisted of narrow well-resolved signals at −78.5~−79.5 ppm related to PhSiO_1.5_ fragments and also narrow signals at −76.8~−77.9 ppm attributed to the PhSiO_1.5_ group in the PhSiO_1.5_-PhSi(OH)O-PhSiO_1.5_ fragments. Earlier, we observed similar upfield signals at −76.0~−77.5 ppm in the spectra of PPSSO with *M_w_* = 1300–16000 [12]. These results were in good accord with those of APCI-MS (See Figure S3 in the SM).

To increase the temperature at which the reaction of **2** was conducted, it was carried out in anisole as a solvent in a molybdenum glass flask at the temperature of 130 °C. The mass spectra of the precipitates were obtained after 5 and 15 min from the beginning of the reaction. The ion peak due to the starting compound **2** was absent even in the former spectrum. The latter one is given as Figure S4 in the SM. In this spectrum, all ions of products being in the spectrum of the precipitate from the mixture obtained after the reaction was implemented in toluene, also in a molybdenum glass flask, at 110 °C for 120 min (Figure S3 in the SM) are present. However, in the spectrum **S3**, the ion peaks of the monohydrate of compound **9** with the 1446 Da nominal mass were maximal in abundance, while all other peaks, except those at *m/z* 996 and 997, were of minor ones. On the contrary, Figure S4 shows several peaks to be abundant (of ions with the 996, 1254, 1291, 1308, 1326, 1446, 1464, 1566, 1584, and 1704 Da nominal masses) (see Figure S4 for the structures of these products and ions.) Two facts are of interest here. The first is the great abundance of peaks of dihydrates in products **7**, **8**, and **9**. Earlier, we showed that two water molecules really added to molecular ions in the mass spectrometer (see Note 6 in the SM). However, the abundances of the corresponding peaks are rather great, and the reaction time was rather short in this case. Hence, the contribution of ions of the incompletely closed **7**, **8**, and **9** formed during the reaction in the flask in the abundances of the corresponding ion peaks cannot be excluded.

The second finding here is that the abundances of ion peaks in this spectrum (and hence the amounts of the corresponding products in the precipitate) of the higher molecular mass products **10** and **11** are greater than in the spectrum of Figure S3. Moreover, ion peaks of the product **12** hydrate and/or its isomer (See Figure S4 in the SM for the structure of **12**) are present in this spectrum, whereas they are absent in Figure 7 and Figure S3. Thus, the reaction of **2** went deeper in this case.

Of interest also is a group of peaks at *m/z* 996, 997, 998, and 999. Earlier, we attributed this ion to the fragmentation of the [M^6^ + H_2_O]^+•^ radical cation with the elimination of three water molecules. However, another fragmentation seems to be possible. All of this is discussed in Note 7 in the SM.

The ^29^Si NMR spectrum of the products of this reaction precipitated for 15 min showed two broad signals at −67.0~−71.0 and −76.5~−80.5 ppm, characteristic of the Ph(HO)SiO and PhSiO_1.5_ fragments, respectively, with the integral intensity ratio of 1:5. This agreed well with the results of the PI APCI mass spectrum.

**Figure 8:**
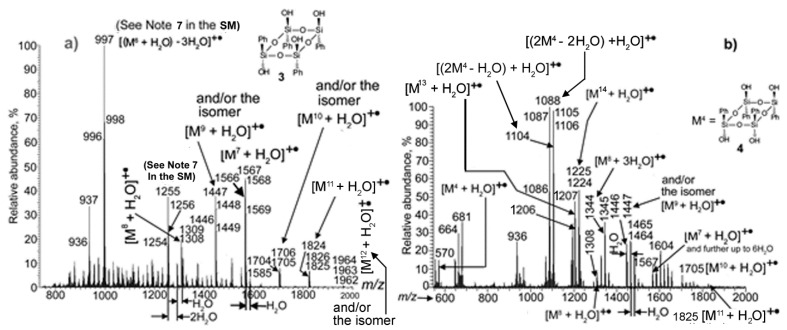
PI APCI mass spectra of precipitates from the reaction mixtures of condensation: (**a**) of compound **3** conducted in a quartz flask in toluene at 110 °C for 120 min and (**b**) of species **4** carried out in a quartz flask in anisole at 150 °C for 120 min (See Note 8 in the SI).

The thermal self-condensation of compound **3** was performed in “pseudo”-equilibrium conditions (under atmospheric pressure) in a quartz flask with toluene as a solvent. The reaction was carried out at 110 °C, and samples of the precipitates were taken for the PI APCI analyses after 10, 20, 40, 60, and 120 min from the beginning of the reaction. The 120 min spectrum is given in Figure 8a. Though the products turned out to be the same, as in the case of compound **2,** the abundances of their peaks and thus their contents in the precipitates were rather different from those in the case of **2**. All spectra did not virtually contain peaks of initial compound **3** (peaks at *m/z* 532 and 570 were present on the background level only). However, peak groups beginning with the peak of the ion with the nominal mass of 996 Da was strongly prevailing in abundance in all the spectra, while the abundances of the other peaks in the spectra were significantly less. This indicated that, in contrast to the case of species **2**, the main product ought to be of a relatively lower molecular mass. With that, in moving from the 10 min spectrum to the 20 min one, the relative abundances of the peaks due to the higher molecular mass products slightly increased while, further, these abundances changed insignificantly (compare the spectra in Figure 8a and Figure S5 in the SM).

**Scheme 2:**
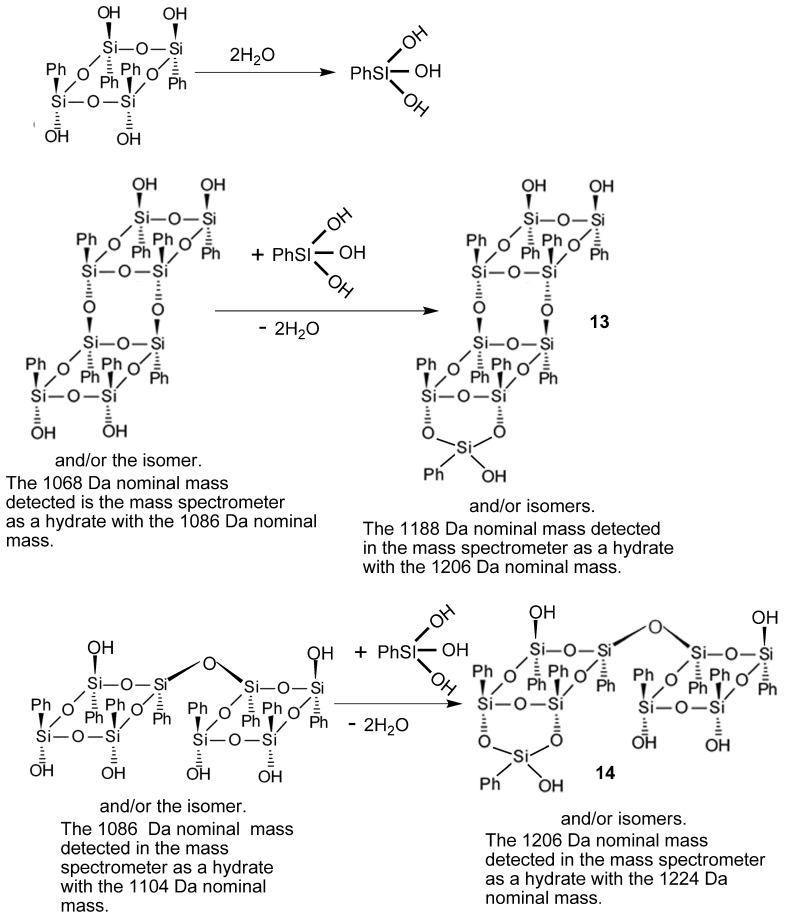
The ways of formation of species **13** and **14** in the course of condensation of **4**.

Since compound **4** was poorly soluble in toluene, its condensation was performed in other solvents. Figure 8b shows the PI APCI mass spectrum of the precipitate from the reaction mixture of the condensation of **4** in anisole at 150 °C for 120 min. The picture here was rather different from that of Figure 8a. Very small abundance peaks of the initial **4** hydrate at *m/z* 570 were detected. The main ions proved to be those of two dimers of **4**; formed with the loss of 2H_2_O and H_2_O, both registered as their hydrates with the nominal masses of 1086 and 1104 Da, respectively. Also present in the spectrum were ions of species **13** and **14** (See Scheme 2), again detected as the hydrates with the nominal masses 1206 and 1224 Da.

The reaction of **4** in acetonitrile went even deeper at a lower temperature. Figure S6a in the SM gives the PI-APCI mass spectrum of the precipitate obtained after the heating of the reaction mixture in a quartz flask at 75 °C for 120 min. The main peaks turned out to be of [M^13^ + H_2_O]^+•^, [M^8^ + 2H_2_O]^+•^, and [M^9^ + 2H_2_O]^+•^ radical cations with the nominal masses 1206, 1326, and 1464 Da, respectively. At that, as it was discussed above, the last two ions, along with contributions from the dihydrates of species **8** and **9** formed during the MS analysis, could contain contributions from the incompletely condensed **8** and **9** from the reaction that occurred in the flask. When the reaction was prolonged up to 240 min, it again went slightly deeper, with peaks of [M^7^ + H_2_O]^+•^ radical cation increasing significantly in abundance. Also, noticeable peaks of [M^10^ + H_2_O]^+•^ and [M^11^ + H_2_O]^+•^ were registered (Figure S6b).

Attempts to carry out the self-condensation of isomer **5** under “pseudo”-equilibrium conditions using different solvents (toluene, anisole, or ditolylmethane) in molybdenum glass in the temperature range of 80–155 °C for 30 h failed. No OSSO were detected by MS. An abundant peak of starting isomer **5** was present only.

## 3. Materials and Methods

### 3.1. Materials

Phenyltrichlorosilane (**1**) was bought from a firm, **abcr**, Karlsruhe, Germany. Commercial grade organic solvents (acetone, acetonitrile, toluene, ethanol, methylene chloride, anisole, ditolylmethane, and tetrahydrofuran) were purified and dried (if necessary) according to conventional procedures. Acetone-*d*_6_ and CD_2_Cl_2_ were used without additional purification.

*cis*-*cis*-*trans*-(Tetrahydroxy)(tetraphenyl)cyclotetrasiloxane (compound **3**) was synthesized by a previously described procedure [28]. The fractional crystallization of a mixture of compounds **3**–**5** from a toluene:diethyl ether solution (1.0:1.0) gave **3**. M.p. 186~188 °C, R_f_ = 0.29 (TLC, eluent, diethyl ether:toluene = 1.0:1.0). ^29^Si NMR (acetone-*d*_6_, δ/ppm): −70.05, −70.20, and −70.35 (all s, ratio 1:1:2). PI APCI-MS, *m*/*z*: 570 ([M^3^ + H_2_O]^+•^) and NI APCI-MS, *m*/*z*: 551 ([M^3^ − H^−^]).

*cis*-*trans*-(Tetrahydroxy)(tetraphenyl)cyclotetrasiloxane (compound **4**) was also synthesized according to the previously described procedure [28]. The fractional crystallization of a mixture of compounds **3**–**5** in a ratio of 0.15:0.50:0.35 from a toluene:diethyl ether solution (1.0:0.25) gave **4** with m.p. 219~222 °C. R_f_ = 0.53 (TLC, eluent, diethyl ether:toluene = 1.0:1.0). ^29^Si NMR (acetone-*d*_6_, δ/ppm): −69.95 (s).

All *trans*-(tetrahydroxy)(tetraphenyl)cyclotetrasiloxane (compound **5**) was obtained according to the procedure reported in [28], including the fractional crystallization of a mixture of compounds **3**–**5** in a ratio of 0.40:0.15:0.45 from a toluene:diethyl ether = 1.0:1.0 solution. M.p. 248~250 °C. R_f_ = 0.64 (TLC, eluent, diethyl ether:toluene = 1.0:1.0). ^29^Si NMR (acetone-*d*_6_, δ/ppm): −70.51 (s). Its structure was earlier confirmed by X-ray diffraction analysis of a single crystal [10].

Compound **6** was obtained by method [34]. M.p. 498 °C, PI APCI-MS, *m*/*z*: 1050 [M^6^ + H_2_O]^+•^, 996 [(M^6^ + H_2_O) − 3H_2_O]^+•^ (see Note 7 in the SM). 

Compound **7** was obtained by a protocol reported in [14]. M.p. 384–386 °C, PI APCI-MS, *m*/*z*: 1566 [M^7^ + H_2_O]^+•^. ^29^Si NMR (CD_2_Cl_2_, δ/ppm): −81.31(s), −84.79 (s).

### 3.2. Measurements

^29^Si NMR spectra were taken on Bruker AV-400 and AV-600 spectrometers (Bruker Corporation, Karlsruhe, Germany) in aceton-*d*_6_ or CD_2_Cl_2_ solutions at 20 °C. 

The PI and NI APCI mass spectra were obtained on a Thermo Finnigan LCQ Advantage tandem dynamic mass spectrometer (Thermo Finnigun, San Jose, CA, USA). The instrument was equipped with an octapole ion trap mass analyzer, a Surveyor MS pump, and a nitrogen generator Schmidlin-Lab., model N2-Mistral-4 (Shmidlin-Lab, Neuheim, Switzerland). Nitrogen was employed as the sheath and auxiliary gases. The temperature of the vaporizer was 400 °C, and the flow rate of acetonitrile was 350 μL min^−1^. The heated capillary temperature was 150 °C; the corona discharge current was 5 μA. Samples were dissolved in acetonitrile and introduced into the ion source through a Reodyne injector with a 5 μL loop. The program Xcalibur, version 1.3 was used for the data collection and treatment.

### 3.3. Methods

#### 3.3.1. The Hydrolysis–Condensation of Phenyltrichlorosilane (**1**)

A solution of compound **1**, 5.2 g (0.025 mol) in 15 mL of acetone, was added at 2~4 °C under stirring to a mixture of water and ice (1:1), 270 g (taken in an amount that was necessary to obtain the required C_HCl_ = 0.26 mol L^−1^ after the complete hydrolysis of **1**). The mixture was then placed into a fridge operated at 4 °C. Products precipitated from the solution in time. The precipitates were isolated after several time periods and their PI APCI mass spectra obtained. Each precipitate was dissolved in diethyl ether; the ether layer was separated, washed with water until the neutral reaction of washing water, and dried over Na_2_SO_4,_ that was then removed. The ^29^Si NMR spectrum was recorded. Ether was evaporated, the precipitate was washed with benzene to remove the impurities including other isomers of (tetrahydroxy)(tetraphenyl)cyclotetrasiloxane which could be present in negligible amounts only according to the ^29^Si NMR spectrum. All the precipitates collected up to 240 h were combined to give 2.52 g of compound **2** (73.6%). M.p. 178~180 °C, R_f_ = 0.05 (TLC, eluent, diethyl ether:toluene = 1:1). ^29^Si NMR spectrum (acetone-*d*_6_, δ/ppm): −69.79 (s). PI APCI-MS, *m/z*: 570 ([M^2^ + H_2_O]^+•^). The molecular structure of compound **2** was earlier confirmed by X-ray powder diffraction [31].

The reactions at the other values of C_HCl_ were implemented in a similar fashion.

#### 3.3.2. General Procedure of the Self-Condensation of Isomers **2**–**4** under “Pseudo”-Equilibrium Conditions (under Atmospheric Pressure)

The corresponding compound (**2**, **3**, or **4**), 55~75 mg, was dissolved in one of the solvents (toluene, anisole, or acetonitrile) taken in the amounts to obtain a 30% solution. A quartz or molybdenum glass flask equipped with a Dean–Stark trap was vacuumized and filled with argon by a triple procedure. The above solution was transferred into it and heated with stirring with a magnetic stirrer at 75, 110, 130, or 150 °C for several h. Samples of the precipitates formed and of the solutions were analyzed at several time points of the reaction by PI APCI-MS and ^29^Si NMR spectroscopy. If there were no changes in the composition of the products, the reaction was stopped, the solvent was removed, and the products were washed with benzene, analyzed, recrystallized, and analyzed again.

## 4. Conclusions

The results obtained showed that the acidity of the water-acetone solutions affected the rate of the hydrolysis–condensation reaction of phenyltrichlorosilane (**1**) and the yields of the products. In the concentration range of C_HCl_ = 0.032~0.26mol L^−1^, **2** was obtained as the main product of reaction whose yield increased with the increase in C_HCl_.

This result differed from the results of the analogous reactions of *m*-tolylSiCl_3,_ *m*-ClPhSiCl_3_, and *α*-naphtylSiCl_3_ published earlier [26] since some products of other types were obtained by these reactions, e.g., *cis-trans*-(tetrahydroxy)(tetra-*m*-chlorophenyl)cyclotetrasiloxane in the case of *m*-ClPhSiCl_3_, and *trans*-1,1,3,3-tetrahydroxy-1,3-di-*α*-naphtyldisiloxane in the case of *α*-naphtylSiCl_3._

For further study, isomers **3**, **4**, and **5** were obtained by rearrangement of isomer **2** when HCl was added to its solution in acetone. 

It was shown that, in “pseudo”-equilibrium conditions (under atmospheric pressure), the self-condensations of isomer **2** in toluene in quartz or molybdenum glass flasks at 110 °C or in anisole in a molybdenum glass flask at 130 °C, and of isomer **3** in toluene in a quartz flask at 110 °C, led to completed Ph-T_8,10,12,14_ and uncompleted cage-like compounds Ph-T_10,12_OSi(HO)Ph. The starting reagents **2** and **3** were already absent after 5 min. In the case of compound **2** in toluene and in a quartz flask, peaks corresponding to compound Ph-T_8_ proved to be prevailing in the PI APCI mass spectra taken at the beginning stages of the reaction, whereas those of Ph-T_10,12,14_ were less abundant. The situation changed in time and the last-mentioned ones became more abundant, with those of Ph-T_12_ becoming predominant. With isomer **3**, the products turned out to be the same, however, Ph-T_8_ remained the major one during the entire reaction. The reaction of **2** in a molybdenum glass flask was characterized by greater amounts of Ph-T_12_ in the mixture and the presence of small amounts of Ph-T_14_. As was underlined above, this might be caused by the catalysis by metal ions present in the molybdenum glass. The move to anisole as a solvent seemed to result in a shift in the equilibrium towards uncompleted cage-like species, their peaks in the PI APCI mass spectrum being more abundant. This appears to be due to a higher temperature of the reaction.

The reactions of isomer **4** were performed in anisole or acetonitrile, since it was poorly soluble in toluene. The main characteristic of the process in anisole was the formation of dimers owing to the loss of one or two molecules of water. They and their derivatives, compounds **13** and **14**, proved to be the main products. In acetonitrile, the reaction went deeper and, along with the dimeric species, compounds Ph-T_10,12_ and incomplete cage-like species formed in significant amounts in spite of the lower reaction temperature. 

Isomer **5** did not enter into the self-condensation reaction. The reason for this appears to be that it dissolved significantly less in the above solvents than **4**, maybe owing to another type of hydrogen bonds in it [10].

## Data Availability

All necessary data are given in the article.

## Supplementary Materials

The following are available online. Notes 1–8. Figure S1: APCI mass spectra of compound **3**: (a) recorded in the PI mode, (b) recorded in the NI mode when the analyte was essentially diluted by acetonitrile. Figure S2: PI APCI mass spectrum of the precipitate from the reaction mixture of the self-condensation of compound **2** carried out in a quartz flask in toluene at 110 °C for 45 min. Figure S3: PI-APCI mass spectrum of the precipitate from the reaction mixture of self-condensation of compound **2** carried out in a molybdenum glass flask in toluene solution at 110 °C for 120 min. Figure S4: PI APCI mass spectrum of a mixture of compounds precipitated from the reaction mixture after the condensation of species **2** was carried out in anisole in a molybdenum glass flask at 130 °C for 15 min. Figure S5: PI APCI mass spectrum of the precipitate from the reaction mixture of the compound **3** condensation conducted in a quartz flask in toluene at 110 °C for 10 min. Figure S6: PI APCI mass spectra of samples of the precipitates from the reaction mixture of compound **4** condensation conducted in acetonitrile in a quartz flask at 75 °C: a) taken after 120 min and b) taken after 240 min.

## Sample Availability

Samples of the compounds are not available from the authors.

## References

[1] Baney R.H., Itoh M., Sakakibara A., Suzuki T. (1995). Silsesquioxanes. Chem. Rev..

[2] Voronkov M.G., Lavrent’ev V.I. (1982). Polyhedral oligosilsesquioanes and their homoderivatives. Top. Curr. Chem..

[3] Abe Y., Gunji T. (2004). Oligo- and polysiloxanes. Prog. Polym. Sci..

[4] Shimojima A., Kuroda K. (2020). Alkoxy- and Silanol-Functionalized Cage-type oligosiloxanes as molecular building blocks to construct nanoporous materials. Molecules.

[5] Li G., Wang L., Ni H., Pittman C.U. (2001). Polyhedral oligomeric silsesquioxane (POSS) polymers and copolymers. J. Inorg. Organomet. Polym..

[6] Li Z., Kong J., Wang F., He C. (2017). Polyhedral oligomeric silsesquioxanes (POSSs) an important building block for organic optoelectronic materials. J. Mater. Chem. C.

[7] Dong F., Lu L., Ha C.-S. (2019). Silsesquioxane-containing hybrid nanomaterials: Fascinating platforms for advanced application. Macromol. Chem. Phys..

[8] Zhang Z.-X., Hao J., Xie P., Zhang X., Han C.C., Zhang R. (2008). A Well-defined ladder polyphenylsilsesquioxane (Ph-LPSQ) synthesizes via a new three-step approach: Monomer self-organization–lyophilization–surface-confined polycondensation. Chem. Mater..

[9] Zhang X., Xie P., Shen Z., Jiang J., Zhu C., Li H., Zhang T., Han C.C., Wan L., Yan S. (2006). Confined synthesis of a *cis*–isotactic ladder polysilsesquioxane by using a π-stacking and H-bonding superstructure. Angew. Chem. Int. Ed. Commun..

[10] Petrova I.M., Buryak A.K., Dolgushin F.M., Peregudov A.S., Klemenkova Z.S., Makarova N.N. (2014). Synthesis of ladder polyphenylsilsesquioxane with columnar structure. Russ. Chem. Bull..

[11] Petrova I.M., Buryak A.K., Peregudov A.S., Strelkova T.V., Kononova E.G., Bushmarinov I.S., Makarova N.N. (2015). Effect of stereoisomerism of (tetrahydroxy)(tetraphenyl)-cyclotetrasiloxanes on the siloxane framework in polyphenylsilsesquioxanes obtained by polycondensation in the presence of layered-architecture compounds. Mendeleev Commun..

[12] Makarova N.N., Peregudov A.S., Klemenkova Z.S., Petrova I.M. (2015). Configuration of cyclolinearpolyphenylsilsesquioxanes as studied by ^29^Si NMR spectroscopy. Mendeleev Commun..

[13] Makarova N.N., Lyakhovetsky Y.I., Petrova I.M., Dolgushin F.M., Ikonnikov N.S., Peregudov A.S., Strelkova T.V., Klemenkova Z.S. (2018). A study of the polycondensation of (tetrahydroxy)(tetraaryl)cyclotetrasiloxanes under equilibrium and non-equilibrium conditions in the presence and absence of montmorillonite. Polymers.

[14] Lee A.S., Choi S.-S., Lee H.S., Baek K.-Y., Hwang S.S. (2012). A new, higher yielding synthetic route towards dodecaphenyl cage silsesquioxanes: Synthesis and mechanistic insights. Dalton Trans..

[15] Choi S.-S., Lee A.S., Hwang S.S., Baek K.-Y. (2015). Structural control of fully condensed polysilsesquioxanes: Ladderlike vs. cage structured polyphenylsilsesquioxanes. Macromolecules.

[16] Chinnam P.R., Gau M.R., Schwab J., Zdilla M.J., Wunder S.L. (2014). The polyoctahedral silsesquioxane (POSS) 1,3,5,7,9,11,13,15-octaphenylpentacyclo-[9.5.1.1^3,9^,1.^5,15^,1^7,13^]octasiloxane (octaphenyl-POSS). Acta Crystallogr. C Struct. Chem..

[17] Tateyama S., Kakihana Y., Kawakami Y. (2010). Cage octaphenylsilsesquioxane from cyclic tetrasiloxanetetrol and its sodium salt. J. Organomet. Chem..

[18] Sugiyama T., Shiba H., Yoshikawa M., Wada H., Shimojima A., Kuroda K. (2019). Synthesis of polycyclic and cage siloxanes by hydrolysis and intramolecular condensation of alkoxysilylatedcyclosiloxanes. Chem. Eur. J..

[19] Oguri N., Egawa Y., Takeda N., Unno M. (2016). Janus-cube octasilsesquioxane: Facile synthesis and structure elucidation. Angew. Chem. Int. Ed..

[20] Uchida T., Egawa Y., Adachi T., Oguri N., Kobayashi M., Kudo T., Takeda N., Unno M., Tanaka R. (2019). Synthesis, structures, and thermal properties of symmetric and Janus “Lantern cage” siloxanes. Chem. Eur. J..

[21] Vogelsang D.F., Dannatt J.E., Maleczka R.E., Lee A. (2018). Separation of asymmetrically capped double-decker silsesquioxanes mixtures. Polyhedron.

[22] Barry B.-D., Dannatt J.E., King A.K., Lee A., Maleczka R.E. (2019). A general diversity oriented synthesis of asymmetric double-decker shaped silsesquioxanes. Chem.Commun..

[23] Kunthom R., Takeda N., Unno M. (2019). Synthesis and characterization of unsymmetrical double-decker siloxane (Basket cage). Molecules.

[24] Kunthom R., Adachi T., Liu Y., Takeda N., Unno M., Tanaka R. (2019). Synthesisof “Butterfly cage” based on double-decker silsesquioxane. Chem. Asian J..

[25] Shimojima A., Kuroda K. (2004). Selective formation of siloxane-based hybrid cages with methylene groups in the frameworks. Chem. Commun..

[26] Petrova I.M., Lyakhovetsky Y.I., Chernyshev V.V., Ikonnikov N.S., Makarova N.N. (2019). A study of the influence of the HCl concentration on the composition and structure of (hydroxy)arylsiloxanes from the hydrolysis–condensation reaction of aryltrichlorosilanes. Molecules.

[27] Klement’ev I.Y., Shklover V.E., Kulish M.A., Tikhonov V.S., Volkova E.V. (1981). Configurational transformation of tetraoxytetraphenylcyclotetrasiloxane. Dokl. Akad. Nauk SSSR.

[28] Makarova N.N., Petrova I.M., Petrovskii P.V., Volkova L.M., Shcherbina M.A., Bessonova N.P., Chvalun S.N., Godovskii Y.K. (2004). Synthesis of new stereoregular 2,4,6,8-tetraphenylcyclotetrasiloxanes with mesogenic groups and the influence of spatial isomerism on the phase state of individual isomers and their mixtures. Russ. Chem. Bull..

[29] Ito R., Kakihana Y., Kawakami Y. (2009). Cyclic Tetrasiloxanetetraols: Formation, isolation, and characterization. Chem. Lett..

[30] Endo H., Takeda N., Unno M. (2017). Stereoisomerization of cyclic silanols. Asian J. Chem..

[31] Dmitrienko A.O. (2015). The Development and Approbation of the Reliability Accuracy Criterion for Geometrical Parameters from the Data of Powder Diffraction. Ph.D. Thesis.

[32] Gabelica V., De Pauw E. (2005). Internal energy and fragmentation of ions produced in electrospray sources. Mass Spectrom. Rev..

[33] Nekrasov Y.S., Ikonnikov N.S., Borisov Y.A., Kiselev S.S., Kornienko A.G., Velezheva V.S., Lykhovetsky Y.I. (2017). Reactivity of *N*-Methylidenemalonats of 3-Arylaminoindoles and *p*-Dimethylamino-*N*-Phenylaniline in the course of their analysis by electrospray ionization mass spectrometry. Int. J. Anal. Mass Spectrom. Chromatogr..

[34] Brown J.F., Vogt L.H., Prescott P.I. (1964). Preparation and characterization of the lower equilibrated phenylsilsesquioxanes. J. Am. Chem. Soc..

[35] Andrianov K.A., Zhdanov A.A., Zavin B.G., Svistunov V.S., Yakushkina S.E. (1971). Polymerization of Methyl(phenyl)cyclotrisiloxanes during Zone melting. Vysokomol. Soedin. B.

