# Supplementary material for: The Influence of HCl Concentration on the Rate of the Hydrolysis–Condensation Reaction of Phenyltrichlorosilane and the Yield of (Tetrahydroxy)(Tetraphenyl)Cyclotetrasiloxanes, Synthesis of All Its Geometrical Isomers and Thermal Self-Condensation of Them under “Pseudo”-Equilibrium Conditions
Source: Molecules. 2021 Jul 20;26(14):4383. doi: 10.3390/molecules26144383 (PMC8306532; doi:10.3390/molecules26144383)
Supplement: Supplementary file 1 [file molecules-26-04383-s001.zip › molecules-1274444-supplementary.pdf]

The influence of HCl concentration on the rate of the hydrolysis–condensation reaction of phenyltrichlorosilane and the yield of tetrahydroxy(tetraphenyl)cyclotetrasiloxanes, synthesis of all its geometrical isomers, and thermal self-condensation of them under "pseudo"-equilibrium conditions.

Irina M. Petrova,[1] Yury I. Lyakhovetsky,[1] Nikolai S. Ikonnikov[1] and Nataliya N. Makarova[1]*

[1]A.N. Nesmeyanov Institute of Organoelement Compounds of the Russian Academy of Sciences, 28 Vavilov St., 119991 Moscow V-334, Russia, Fax: +7(499) 135 5085; impetr2013@yandex.ru (I.M.P.); yulyakh@ineos.ac.ru (Y.I.L.); ikonns@ineos.ac.ru (N.S.I.); nmakar@ineos.ac.ru (N.N.M.).

*Correspondence: *nmakar@ineos.ac.ru*; Tel.: +7(499)700 5870, ext. 1180

Supplementary Materials (SM)

Note 1. In our experiments, HCl formed due to the hydrolysis of $PhCl_3$ and its concentration was achieved by the corresponding dilution of the reaction mixture. However, at dilution the concentration of $PhSi(OH)_3$ formed by the hydrolysis decreased. This certainly influenced the rate of the reaction. However, the concentration of the silane decreased in the course of the reaction, whereas the concentration of HCl remained constant equal to the triple value of the initial concentration of $PhSi(OH)_3$. This allowed us to consider that the main effect upon the reaction was exerted by the change in the HCl concentration. Thus, ascribing the alterations of the rate of the reaction and the yields of the products to the changes in the HCl concentration appears to be practically correct.

Note 2. One may suggest that a water molecule was initially in compounds **2** and **3**. However, powder X-ray diffraction analysis of **2** and also X-ray structural analysis of compound **5** showed no water to be present and thus, water was added in the mass spectrometer. This conclusion is in a good concordance with the observations in our previous studies where we also showed that water was added to the analyte molecules during the mass spectral analyses (see refs. [13] and [26] in the main text of the article, and SI to them). However, we again cannot tell whether the hydrate of $[M^2]^{+\bullet}$ was formed or the addition occurred with the cleavage of a Si-O bond.

Note 3. Certainly, the 1086 Da ion could be interpreted as $[(M^2)_2 - H_2O]^{+\bullet}$ but taking in account Note 2, presenting it as $[(M^2)_2 - 2H_2O) + H_2O]^{+\bullet}$ appears to be more proper. The

same involving the addition of a water molecule in the mass spectrometer is valid for the 1068 and 1104 Da ions.

Note 4. The analysis of mass chromatograms showed that probably, radical anion $[M^2]^{\bullet-}$ was also present. Besides the ions mentions in the text, ions with masses by 43 and 86 Da higher than that of the $[M^2 - H]^-$ were found in the spectra (Figure 4c and d). They could be formed due to the addition to this ion of the $MeCO^\bullet$ radicals formed via dissociation of the $[MeCONH_2]^-$ anion since acetamide was probably present in acetonitrile as an impurity.

Note 5. Molibdenum glass contains impurities of metal ions, while quartz does not. To avoid the possibility of catalysis by these ions, the first experiments were conducted in quartz flasks.

Note 6. Further investigations showed that when pure **6** was dissolved in a great amounts of acetonitrile, it was registered in the PI APCI mass spectrum as dihydrate $[M^6 + 2H_2O]^{+\bullet}$, a very small amounts of trihydrate $[M^6 + 3H_2O]^{+\bullet}$ being also present.

Note 7. Peaks of the ions with nominal mass of 996 and 1254 Da were present in all PI APCI mass spectra of the precipitates from reaction mixtures of **2** **(**Figure 7, S3, and S4.), We thought these ions to be formed due to fragmentations of the radical cations of the compound **6** and **8** hydrates, $[M^6 + H_2O]^{+\bullet}$and $[M^8 + H_2O]^{+\bullet}$, respectively, with elimination of $3H_2O$. However these processes involve abstraction of four hydrogen atoms from phenyl rings that seems to be not easy. It induced us to consider another possible fragmentation processes, this one of the $[M^6 + H]^+$and $[M^8 + H]^+$ cations or $[M^6]^{+\bullet}$ and $[M^8]^{+\bullet}$ radical cation:

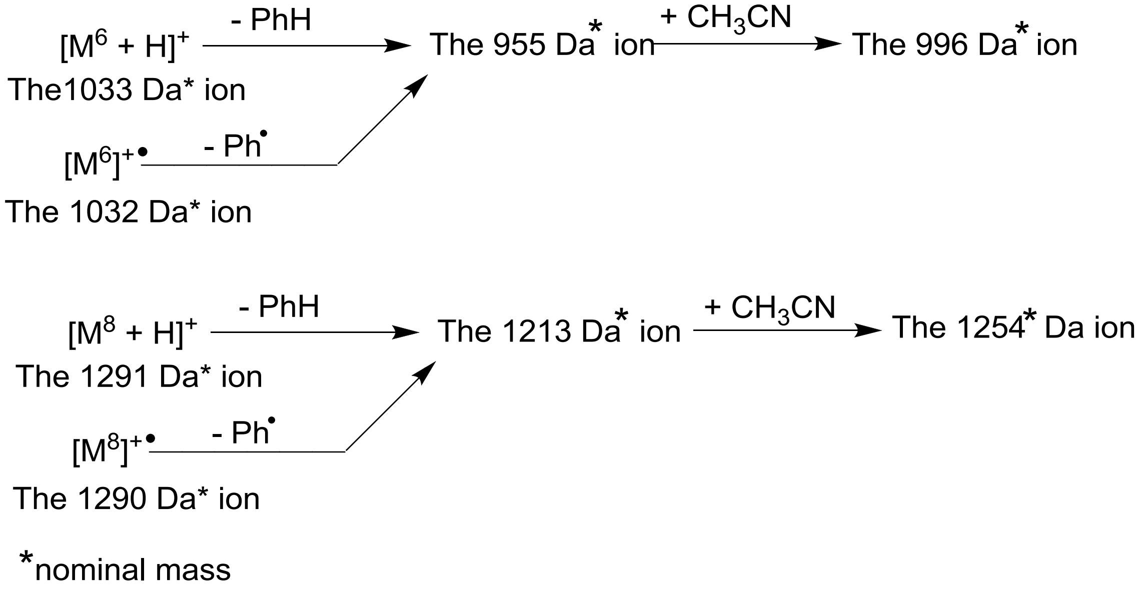

This scheme, as though, is supported by two findings: the ms^2 spectrum of the 996 Da ion showed the loss the 41 Da mass fragment to give the 955 Da ion. When a sample was

dissolved in methanol instead of acetonitrile before the introduction in the mass spectrometer, no 996 Da ion was detected.

However, the peaks of $[M^8 + H]^+$, $[M^6]^{+\bullet}$ (at *m/z* 1033 and 1032), and that at *m/z* 955 were virtually unpresent in the spectrum of pure **6** (were present there as ones of extremely low abundances, on the background level), while the *m/z* 996 peak was abundant (Figure 5). Moreover, peaks at m/z 1213 were absent or almost absent in the spectra of the precipitates from the reaction mixtures of compound **2** condensation. All of that speaks that the origin of the *m/z* 996 and 1254 ions remains still uncertain, and this problem requires further investigation.

The same considerations appears to be valid for the PI APCI mass spectrum of pure **6** (Figure 5).

Note 8. In those cases when the abundances of higher *m/z* peaks of the products were significantly greater than the values that should be according to the isotope distribution patterns, the contribution in the abundances from protonated species seemed to be possible.

Additional mass spectra

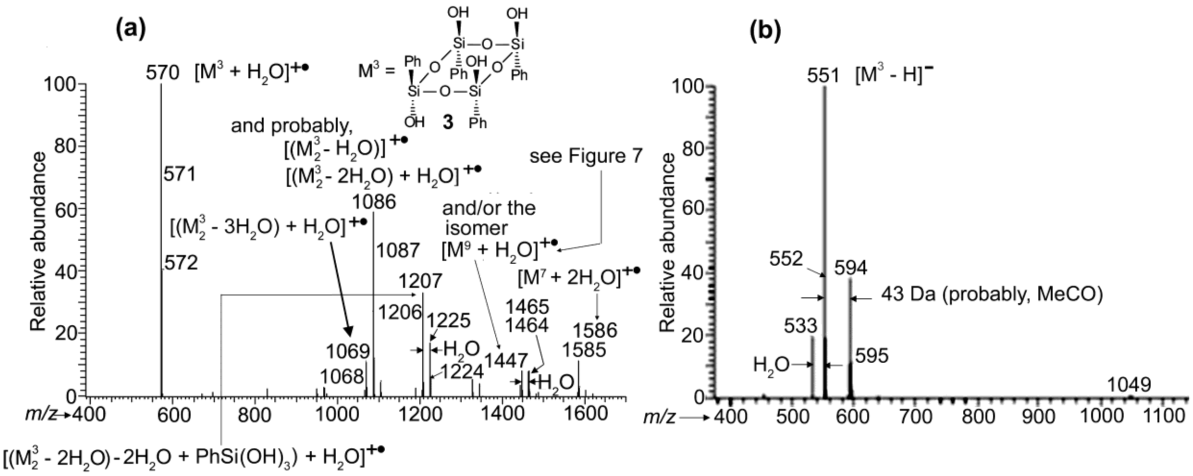

Figure S1. APCI mass spectra of compound **3:** **(a)** recorded in the PI mode, **(b)** recorded in the NI mode when the analyte was essentially diluted by acetonitrile.

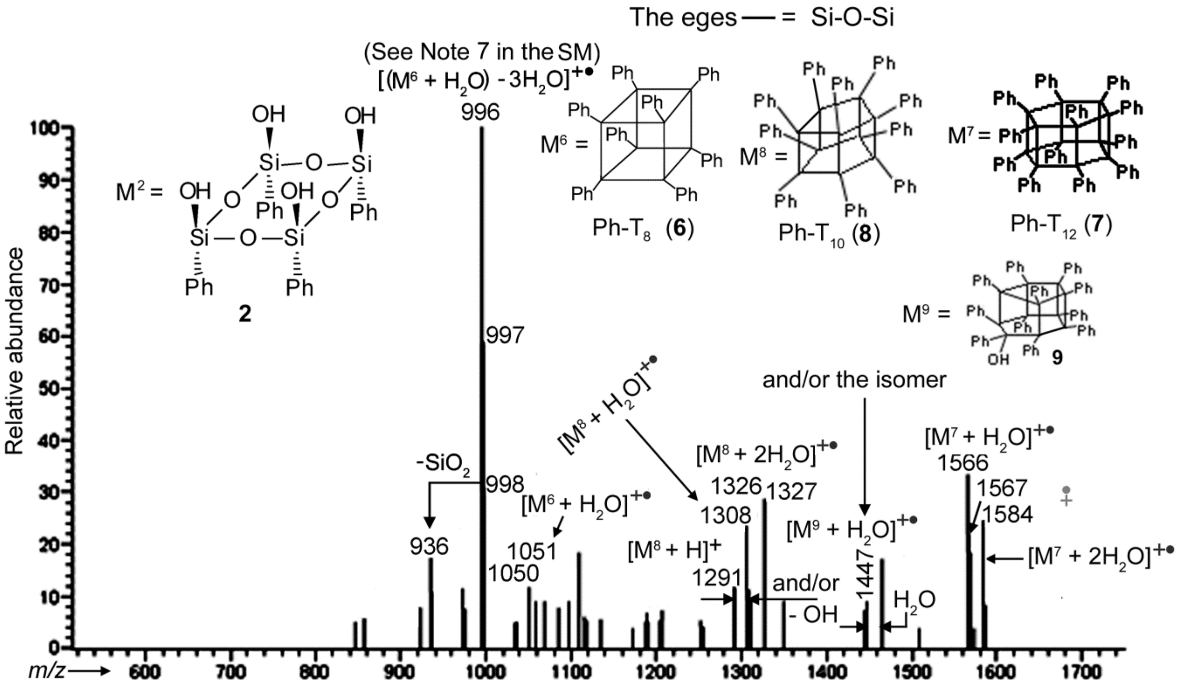

Figure S2. PI APCI mass spectrum of the precipitate from the reaction mixture of the self-condensation of compound **2** carried out in a quartz flask in toluene at 110 ^{O}C for 45 min.

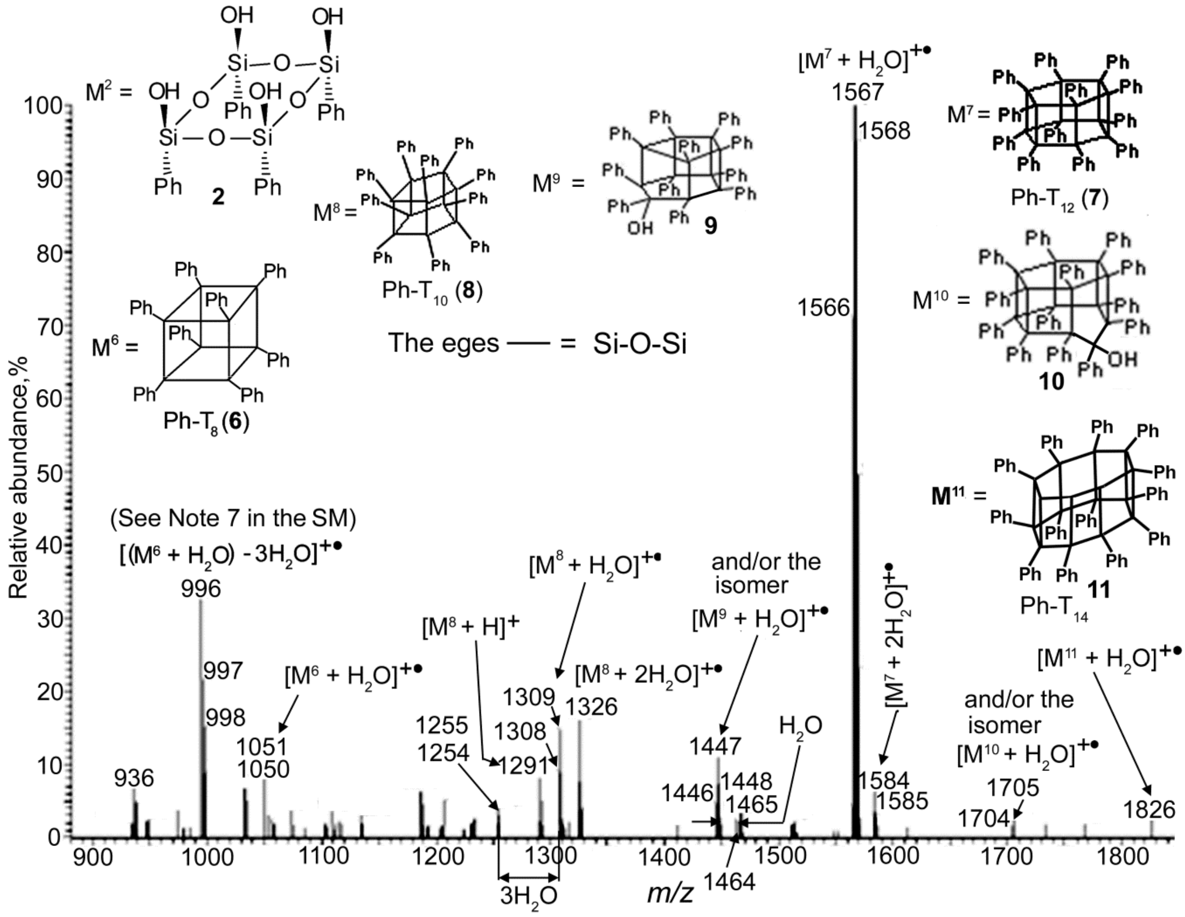

Figure S3. PI-APCI mass spectrum of the precipitate from the reaction mixture of self-condensation of compound **2** carried out in a molybdenum glass flask in toluene solution at 110 ^{0}C for 120 min.

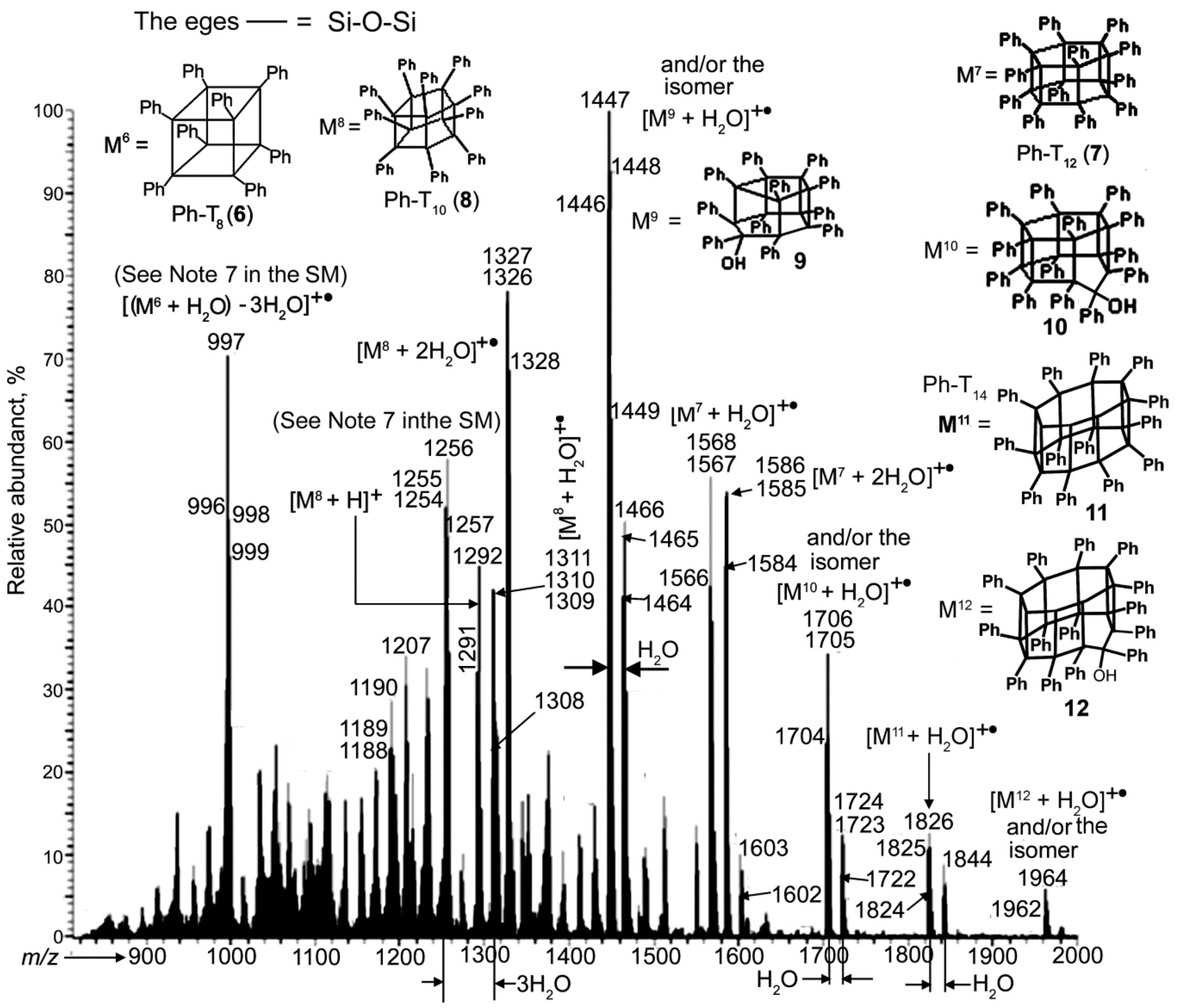

Figure S4. PI APCI mass spectrum of a mixture of compounds precipitated from the reaction mixture after the condensation of species **2** was carried out in anisole in a molybdenum glass flask at 130 ^{0}C for 15 min.

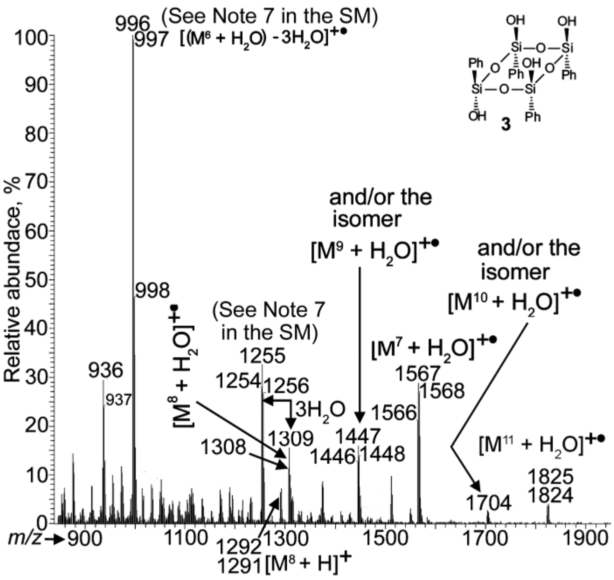

Figure S5. PI APCI mass spectrum of the precipitate from the reaction mixture of the compound **3** condensation conducted in a quartz flask in toluene at 110 ^{O}C for 10 min.

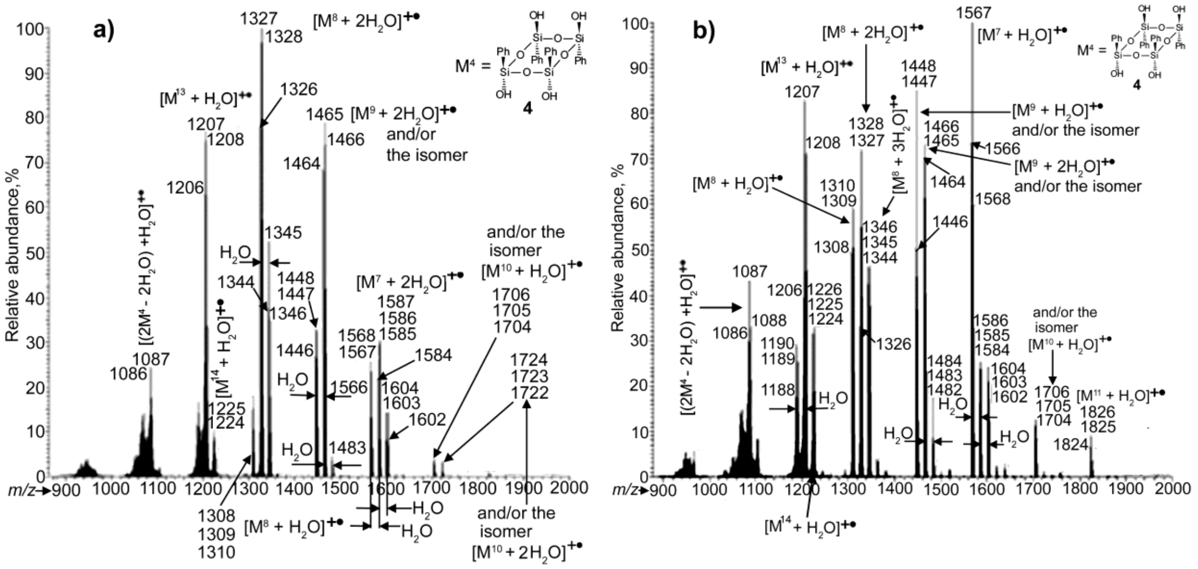

Figure S6. PI APCI mass spectra of samples of the precipitates from the reaction mixture of compound **4** condensation conducted in acetonitrile in a quartz flask at 75 ^{O}C: **a**) taken after 120 min and **b)** taken after 240 min.
